# Protection of the biconcave profile of human erythrocytes against osmotic damage by ultraviolet-A irradiation through membrane-cytoskeleton enhancement

**DOI:** 10.1038/cddiscovery.2017.40

**Published:** 2017-07-17

**Authors:** Cunbo Li, Zheming Li, Shuang Xun, Pengchong Jiang, Rui Yan, Mincai Chen, Fen Hu, Romano A Rupp, Xinzheng Zhang, Leiting Pan, Jingjun Xu

**Affiliations:** 1 Key Laboratory of Weak-Light Nonlinear Photonics, Ministry of Education, TEDA Institute of Applied Physics and School of Physics, Nankai University, Tianjin, China; 2 Department of Chemistry, University of California, Berkeley, Berkeley, CA, USA; 3 Department of Blood Transfusion, PLA 307 Hospital, Beijing, China; 4 The 2011 Project Collaborative Innovation Center for Biological Therapy, Nankai University, Tianjin, China; 5 Collaborative Innovation Center of Extreme Optics, Shanxi University, Taiyuan, Shanxi, China

## Abstract

To perform various physiological functions, erythrocytes possess a unique biconcave shape provided by a special architecture of the membrane-skeleton system. In the present work, we use a simple irradiation method to treat human erythrocytes with 365 nm ultraviolet-A (UVA) light at the single-cell level *in vitro*. Depending on the irradiation dose, UVA show protection of the biconcave profile against the detrimental action of distilled water. This protective effect can also be confirmed for saponin that damages the membrane-skeleton by vesiculation and pore formation. Interestingly, at two irradiation doses of UVA pretreatment, erythrocytes still seem to exhibit cell viability as tested by trypan blue assay even if distilled water or saponin is added. The oxidants hydrogen peroxide and cumene hydroperoxide partly simulate the protective effects. Taken together, these results demonstrate that 365 nm UVA irradiation can protect the biconcave profile of human erythrocytes through membrane-skeleton enhancement associated with a production of oxidants.

## Introduction

Ultraviolet (UV) light has an important role for life on earth. Long-wavelength ultraviolet-A (UVA, 320~400 nm) light, having deeper penetration and lower phototoxicity than short-wavelength UV light (200~320 nm), is known to have various positive effects for human health.^1–6^ For instance, whole-body UVA irradiation results in a significant drop of blood pressure owing to release of nitric oxide from intracutaneous photolabile nitric oxide derivates.^7,8^ UVA therapy is also an effective treatment for acute graft-versus-host disease of the skin.^9^ A recent study identified a UVA-specific phototransduction pathway in melanocytes that induces rapid calcium mobilization via transient receptor potential A1 ion channels.^10^ Several physiological and pathological effects could be traced back to the generation of reactive oxygen species (ROS) by UVA irradiation.^11–13^

Human erythrocytes are well known for their unique biconcave shape owing to a special organization of the membrane-skeleton system.^14^ This particular profile maximizes the surface to volume ratio and thus may expedite diffusion and exchange.^15^ Recently, Uzoigwe^16^ pointed out that the biconcave shape might also optimize flow properties of blood in large vessels by maximization of laminar flow and minimization of platelet scatter, which suppresses atherogenic activity in large vessels. On the other hand, loss of the biconcave shape was associated with some diseases including hereditary spherocytosis and sickle-cell anemia.^17,18^ Stored erythrocytes lose integrity of shape and become more fragile by aging, which results in hemolysis that may contribute to complications for transfusion.^19,20^

Owing to reduction of ozone in the stratosphere, more UVA radiation may reach the ground. This increase of irradiation raised some concerns about human health, because UVA light penetrates down to the dermal layer of the skin and thus, can directly affect erythrocytes inside the capillary. All these reasons have motivated us to continue investigating the effect of UVA irradiation on the shape of human erythrocytes *in vitro*.

## Results

### Effects of UVA preirradiation on the shape of human erythrocytes against distilled water

**Figure 1:**
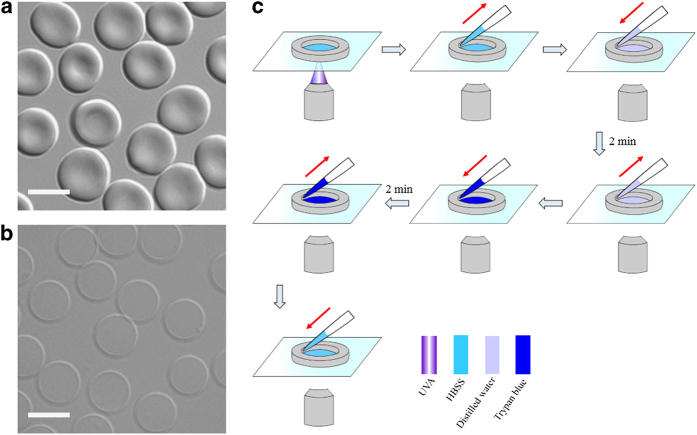
Schematic diagram of the experimental procedure for treatment with UVA, distilled water and trypan blue in human erythrocytes. (**a**) Normal erythrocytes with typical biconcave shape visualized by differential interference contrast (DIC). (**b**) Ghost erythrocytes resulting from distilled water. (**c**) The diagram shows the sequence of application of UVA, distilled water, and trypan blue. Scale bars: 7 *μ*m.

Normal human erythrocytes typically have a biconcave profile as shown in Figure 1a. Distilled water easily permeates into erythrocytes and damages the integrity of the membrane-skeleton system, thereby generating so-called ghosts (Figure 1b). As erythrocytes have neither nucleus nor mitochondria, we used trypan blue exclusion as a suitable assay to test for cell viability. Based on an inverted fluorescence microscope, erythrocytes were irradiated by UVA at an intensity of 2.4 W/cm^2^ for an irradiation time *t*_R_=1, 2, 3, 5, 6, 11 and 20 min, respectively (Supplementary Figure S1). Then, 0.2% (w/v) trypan blue and distilled water were added in turn. Figure 1c shows the detailed procedure of the experiments.

**Figure 2:**
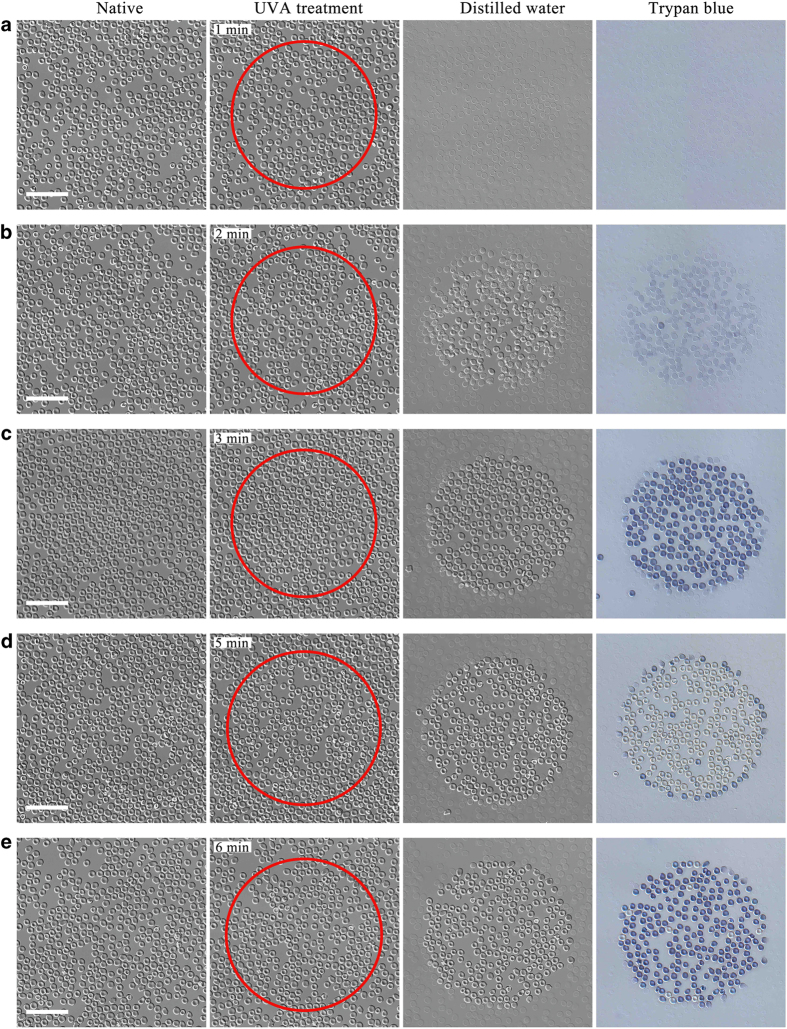

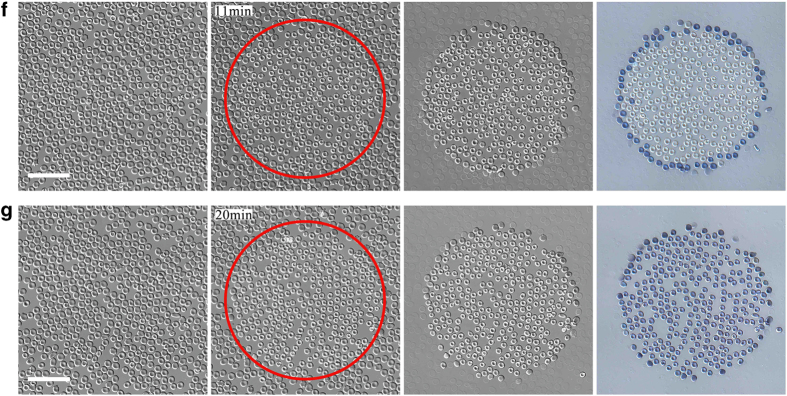
UVA irradiation significantly protects the biconcave shape of human erythrocytes against distilled water. Cells were irradiated by 2.4 mW/cm^2^ UVA for *t*_R_=1 min (**a**), 2 min (**b**), 3 min (**c**), 5 min (**d**), 6 min (**e**), 11 min (**f**) and 20 min (**g**), respectively. Images in the first column correspond to the native erythrocytes visualized by DIC. The second column shows pictures of erythrocytes after treatment with UVA for different times (inside of the red circle). Data in the third column indicate that UVA irradiation abolishes ghosts generated by distilled water in a dose-dependent manner. The images in the fourth column show that cell viability exhibits a periodic change instead of monotone change with increasing irradiation dose as tested by trypan blue stain. Scale bars: 50 *μ*m.

Distilled water still turns cells into ghosts at *t*_R_=1 min UVA pretreatment as shown in Figure 2a. However already, at *t*_R_=2 min, most cells do not disappear anymore albeit, the biconcave shape is not kept well (Figure 2b). For *t*_R_⩾3 min, the biconcave shape is finally maintained as shown in Figure 2c-g. Thus there is a threshold dose above which UVA irradiation protects the biconcave shape. Trypan blue exclusion assay confirms for *t*_R_=5 min and 11 min that UVA not only protects the native shape of erythrocytes from attack by distilled water, but seems to maintain cell viability as well (Figure 2d and f). Interestingly, with increasing irradiation dose from *t*_R_=3 min to 20 min, cell viability does not change monotonically but seems to change periodically.

### Observation of UVA-induced phototoxic effect on erythrocytes

**Figure 3:**
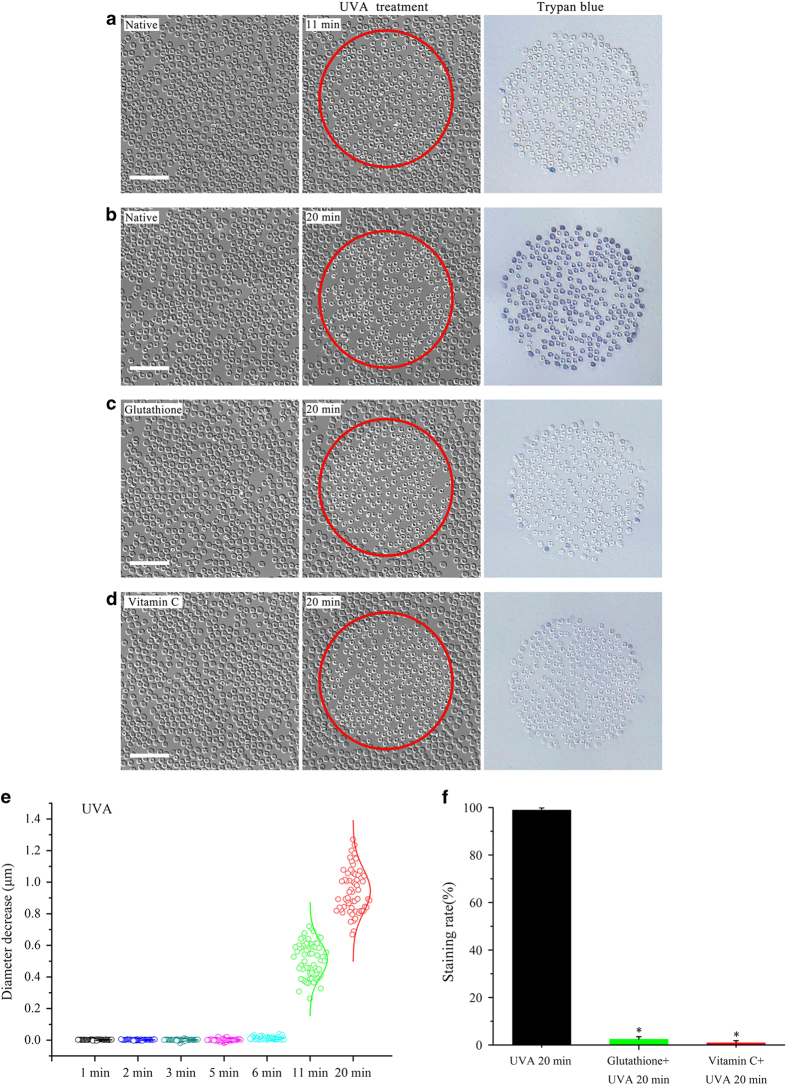
Phototoxic effect of UVA irradiation on human erythrocytes is blocked by glutathione and vitamin C. Trypan blue could not directly stain erythrocytes after treatment of UVA for 11 min (**a**), however, all UVA-irradiated cells were marked by trypan blue at *t*_R_=20 min (**b**). Erythrocytes were preincubated with the antioxidants glutathione (3 mM) and vitamin C (3 mM) for 1 h, respectively. As a consequence, trypan blue was not detected anymore in the presence of glutathione (**c**) and vitamin C (**d**) at *t*_R_=20 min, indicating the efficient inhibitory effect of antioxidants on phototoxic effect resulting from UVA. (**e**) Summary of the diameter decrease of erythrocytes induced by UVA irradiation at *t*_R_=1, 2, 3, 5, 6, 11 and 20 min, respectively (*n*=60 cells for each group from three independent experiments). (**f**) Statistics of staining rate of UVA-treated erythrocytes in the presence of glutathione and vitamin C at *t*_R_=20 min. Data are expressed as mean±S.E.M. and analyzed using Student's *t*-test (*n*=200 cells for each group from three independent experiments). *P<0.01, statistically significant, compared with the UVA 20 min group. Scale bars: 50 *μ*m.

To test whether UVA irradiation has a direct phototoxic effect on erythrocytes, we applied trypan blue to examine cell viability immediately after UVA treatment. It turned out that trypan blue could not permeate into erythrocytes for *t*_R_ below 11 min as shown in Figure 3a. At *t*_R_=20 min, cells were directly stained by trypan blue (Figure 3b). On average no obvious effect was found on the profile of erythrocytes for *t*_R_⩽6 min. In contrast, there is an average shrinking of the cell diameter by 0.5 μm and 0.9 μm at *t*_R_=11 and 20 min, respectively (Figure 3e).

To check whether UVA-induced phototoxicity is caused by ROS, erythrocytes were incubated with two antioxidants glutathione (3 mM for 1 h) and vitamin C (3 mM for 1 h) prior to UVA irradiation, respectively. Both antioxidants show a significant inhibitory effects on uptake of trypan blue after 20 min of UVA irradiation (Figure 3c and d), reducing the staining rate from 94.7% to 2.4% (glutathione) and 0.9% (vitamin C), respectively (Figure 3f). Thus, prolonged UVA irradiation induces phototoxicity by ROS production.

### Effects of oxidants on erythrocytes

**Figure 4:**
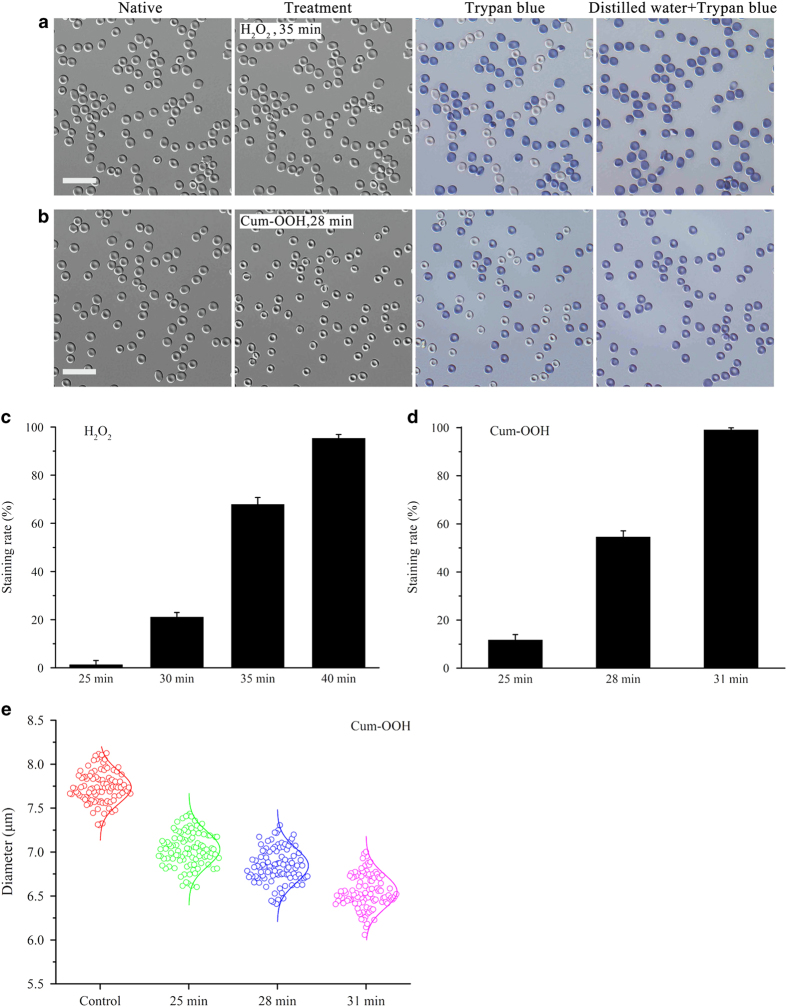
H_2_O_2_ and Cum-OOH partly simulate the protective effect of UVA on the profile of human erythrocytes against distilled water. (**a**) and (**b**) show the typical results of the effects of H_2_O_2_ (10 mM for 35 min) and Cum-OOH (0.6 mM for 28 min) on the shape of erythrocytes against distilled water visualized by DIC. H_2_O_2_ and Cum-OOH preserve the shape of erythrocytes from distilled water, but cannot stop trypan blue staining of the cells. More notably, Cum-OOH rather than H_2_O_2_ lead to a shrinkage of the erythrocytes (**c**) and (**d**) are the statistical results of the staining rate of the cells after treatment with H_2_O_2_ and Cum-OOH (*n*=200 cells for each group from three independent experiments). (**e**) Statistic data on the erythrocytes diameter after treatment of 0.6 mM Cum-OOH for 0 (control), 25, 28 and 31 min, respectively (*n*=60 cells for each group from three independent experiments). Scale bars: 30 *μ*m.

To further investigate the role of ROS in erythrocytes, we used cytosol-soluble hydrogen peroxide (H_2_O_2_) and membrane-soluble cumene hydroperoxide (Cum-OOH)^21^ to simulate the behavior of UVA *in vitro*, respectively. First, erythrocytes were preincubated with 10 mM H_2_O_2_ and 0.6 mM Cum-OOH with different time. Then, typan blue and distilled water was added in turn. It was found that H_2_O_2_ and Cum-OOH effectively protect the erythrocytes profile from distilled water at incubation times larger than ~25 min, which is similar to the observed effect of UVA irradiation. In contrast to UVA irradiation, erythrocytes were all stained by trypan blue after application of distilled water under treatment of oxidants. Typical results are presented for cells with treatment of H_2_O_2_ and Cum-OOH as shown in Figure 4a and b. Both oxidants directly cause cells death for incubation times >40 min. Specifically, the staining rate of H_2_O_2_-treated cells was about 1% for 25 min, 21% for 30 min, 68% for 35 min and 96% for 40 min, respectively (Figure 4c). On the other hand, erythrocytes treated by Cum-OOH exhibits higher staining rate which was ~12% for 25 min, 55% for 28 min and 99% for 31 min, respectively (Figure 4d). Statistics indicates that Cum-OOH causes a significant shrinkage of erythrocytes profiles with increasing incubation time, reducing the average diameter from 7.7 μm (native) to 7.0 μm (25 min), 6.8 μm (28 min), and 6.5 μm (31 min), respectively (Figure 4e). These results are again similar to the effect observed for UVA irradiation (Figure 3e). However, H_2_O_2_ did not have such an obvious effect on the diameter of erythrocytes. In brief, H_2_O_2_ and Cum-OOH can only partly simulate the protective effect of UVA on the erythrocytes shape against attack by distilled water, and indicates that the UVA-induced protective effect is associated with generation of ROS.

### Effects of UVA irradiation on erythrocytes shape against saponin

**Figure 5:**
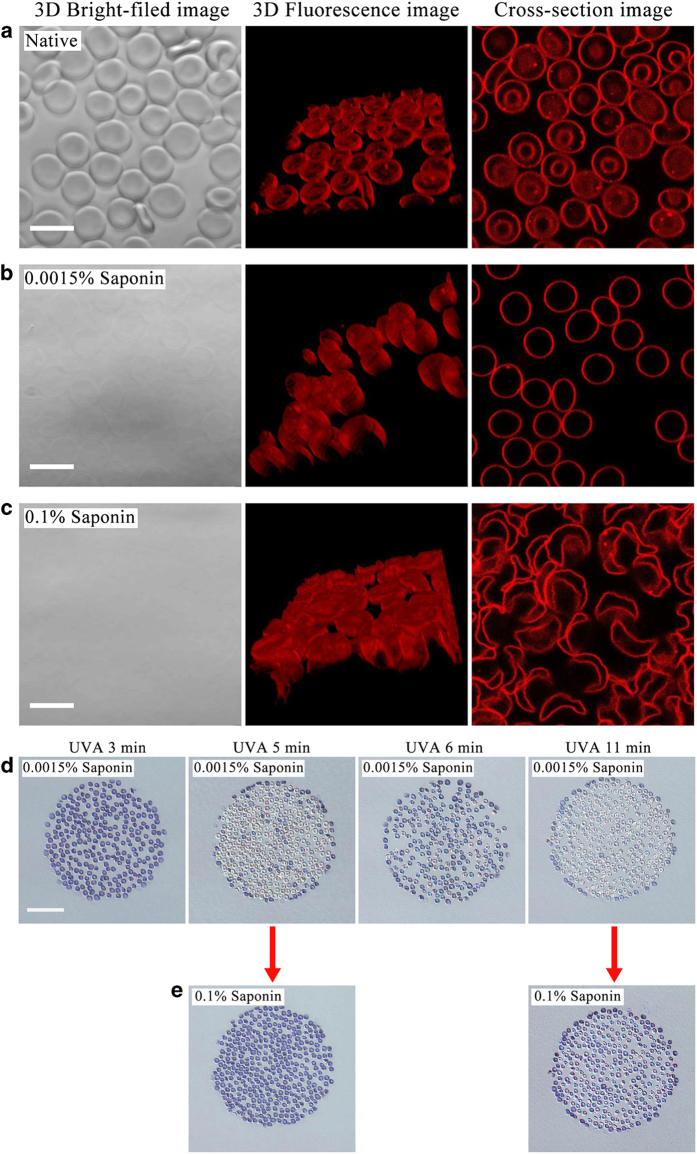
UVA irradiation also protects the erythrocytes profile against saponin. Confocal Laser Scanning Microscopy of membrane lipid stained by nile red revealed the shape change of erythrocytes after treatment with 0 (**a**), 0.0015% (**b**), and 0.1% (**c**) saponin, respectively. (**d**) Trypan blue staining results of UVA-pretreated erythrocytes show a periodic change of cell viability after application of 0.0015% saponin. (**e**) Re-addition of 0.1% saponin induce cell staining by trypan blue at *t*_R_=5 and 11 min. White scale bars: 10 *μ*m; black scale bar: 50 *μ*m.

Saponin, the steroid or triterpenoid glycosides, can interact with the cell membrane because of the affinity of the aglycone moiety for membrane cholesterol,^22,23^ which induces vesiculation and pore formation in cell membranes, even resulting in disruption of the membrane-skeleton system at high concentration. We used saponin (S4521, Sigma-Aldrich, St Louis, MO, USA) to further investigate whether the UVA-induced protective effect on erythrocytes shape is associated with the membrane-skeleton system. First, we marked the membrane using the fluorescent probe nile red to observe the change of the shape induced by lower and higher concentration of saponin, respectively. 3D confocal images show again the native erythrocytes with biconcave profile (Figure 5a). Application of 0.0015% saponin (w/v) not only causes erythrocytes death (hemolysis), but also changes the shape into spheres because dead cells lose control of the membrane-skeleton system against homogeneous surface tension (Figure 5b). In contrast, erythrocytes exhibit extremely irregular shape after treatment with 0.1% saponin, indicating serious damage to the membrane-skeleton system (Figure 5c). These results suggest that saponin destroyed the membrane-skeleton system of erythrocytes in a dose-dependent manner.

Pretreating erythrocytes with UVA for *t*_R_=3, 5, 6 and 11 min, respectively, we find again a significant protective effect with respect to the biconcave shape of erythrocytes regardless of the concentration of saponin (Figure 5d and e). The viability of UVA-pretreated cells tested by trypan blue also exhibits a periodic change after application of 0.0015% saponin (Figure 5d), which is similar to the findings using distilled water (Figure 2). Specifically, although UVA fails to prevent cell death induced by 0.0015% saponin at *t*_R_=3 min, the biconcave profile is completely maintained as compared with the spherical one. This further indicates that the strengthening of the membrane-skeleton system result from UVA irradiation.

### Effects of UVA irradiation on the deformability of erythrocytes

**Figure 6:**
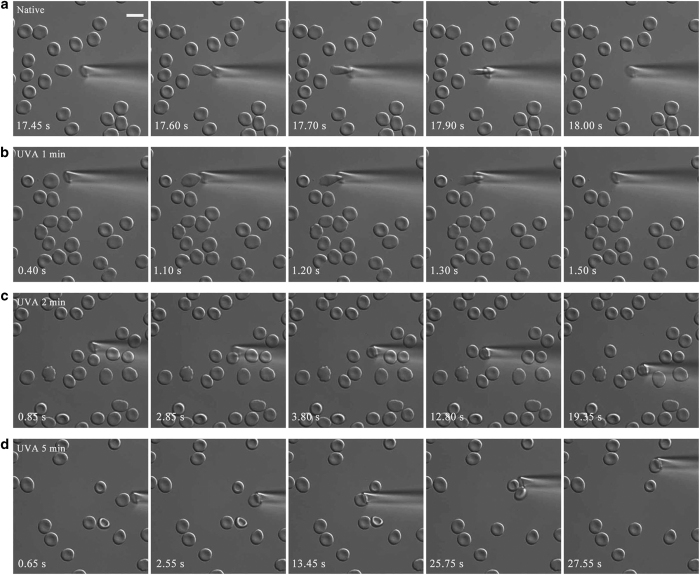
The deformability of erythrocytes decreases with rising UVA irradiation dose. A micropipette was used to measure the deformation of erythrocytes based on siphonage. Cells were irradiated with UVA for 0 (**a**), 1 (**b**), 2 (**c**) and 5 min (**d**), respectively. Scale bars: 10 *μ*m.

We further evaluated the deformability of erythrocytes after treatment with UVA using a micropipette aspiration technique based on siphonage. Data show that native erythrocytes are inspired into micropipette easily and rapidly, indicating the excellent deformability and flexibility (Figure 6a; Supplementary Movie S1). At *t*_R_=1 min, UVA has no evident effect on the deformability (Figure 6b and Supplementary Movie S2). However, at *t*_R_=2 min and 5 min, erythrocytes could not be inspired into micropipette even though the duration of aspiration was longer, suggesting the loss of deformability (Figure 6c and d; Supplementary Movie S3 and S4). These results propose that UVA irradiation results in a loss of deformability in a dose-dependent manner.

## Discussion

Distilled water can easily lyse the erythrocytes by destroying the membrane-skeleton system because of hypotonic swelling. Saponin has a disruptive effect on the membrane-skeleton system owing to vesiculation and pore formation.^22,23^ Our experience is that 365 nm UVA irradiation protects the biconcave shape of human erythrocytes against the detrimental effect of distilled water (Figure 2) as well as of saponin (Figure 5) in a dose-dependent manner. UVA not only preserves the shape of erythrocytes from distilled water or saponin, but also seems to maintain cell viability as probed by trypan blue exclusion. Normally, one would expect a monotone response of the cell with increasing irradiation dose. However, we find that the cell viability of UVA-pretreated erythrocytes changes periodically with increasing irradiation dose after application of distilled water (Figure 2c–g) or saponin (Figure 5d). In brief, our data clearly show an interesting dose-dependent action of UVA on the protection of the biconcave shape of human erythrocytes.

As ROS has an important role in UVA-induced cellular responses,^11–13^ we investigated whether ROS is involved in the protective effect of UVA. On the one hand, results show that both antioxidants, glutathione and vitamin C, evidently inhibit trypan blue uptake at a UVA irradiation time of 20 min (Figure 3c and d). On the other hand, both potent oxidants, the cytosol-soluble H_2_O_2_ and the membrane-soluble Cum-OOH,^21^ completely protect the profile of the erythrocytes against distilled water. Unfortunately, cells were all marked by trypan blue after treatment with distilled water (Figure 4a and b), indicating that oxidants can only partly simulate the protective effect of UVA on the erythrocytes profile. In addition, Cum-OOH rather than H_2_O_2_ leads to a shrinkage of erythrocytes (Figure 4e), which is similar to the effect of UVA (Figure 3e). It indicates that the effect of Cum-OOH is more similar to the one of cytosol-soluble H_2_O_2_. It was reported that Cum-OOH could induce shrinkage of erythrocyte own to oxidation of unsaturated fatty acid in cell membrane.^24^ This shrinkage was associated with deformability reduction,^21^ membrane protein aggregation,^25^ and transmembrane electric field variation.^26^ Shape and size are important for erythrocytes to maintain their functions. For instance, shrinkage of erythrocyte resulted from Rac GTPases deficiency was coupled with significant decrease of deformability and increase of hemolysis.^27^ Similarly, increased plasmatic viscosity induced by shrinkage during erythrocyte aging led to deformability attenuation,^28^ and further compromised the efficiency of tissue oxygen delivery.^29^

It is demonstrated that visible light irradiation can induce various biological effects by generation of ROS.^30,31^ We also irradiated erythrocytes at 488 nm (2.3 W/cm^2^) and 546 nm (2.2 mW/cm^2^) for 20 min, respectively, followed by addition of distilled water. However, no protective effect on erythrocytes profile was observed at these wavelength (data not shown), thus pointing out the exceptional position of UVA light. In addition, it is known that human erythrocytes exhibit extreme deformability as they are subject to huge reversible shear stress while squeezing through narrow capillaries of the microcirculation during their 120-day lifespan. However, our results showed that UVA irradiation could decrease the deformability of erythrocytes (Figure 6 and Supplementary Movie), indicating that UVA irradiation is a double-edged sword between membrane-skeleton enhancement and deformability. Furthermore, we must admit that UVA irradiation should have an effect on the viability of erythrocytes although trypan blue could not stain the cells in some cases. We believe that these results are interesting and will attract much attention in some research field including blood.

**Figure 7:**
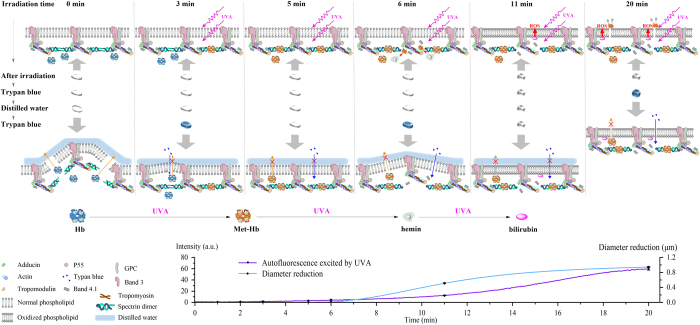
Schematic drawing of the proposed mechanism of the periodically protective effect of UVA on the erythrocytes morphology. The purple curve shows UVA-induced autofluorescence intensity as a function of irradiation time. The blue curve indicates the values of diameter reduction resulting from UVA as a function of irradiation time.

With these data, our next step was to try to discuss the detailed mechanisms of UVA-induced protective effect on human erythrocytes. First, it is not difficult to know that at least two factors should underlie the periodical changes of trypan blue staining induced by UVA with monotone increasing of the irradiation dose. Second, the biconcave shape of human erythrocytes apparently depends on a special organization of the membrane-skeleton system. Thus, according to our results and the reports of other researchers, we explain the protective mechanisms related to membrane-skeleton system as shown in Figure 7.

It has been suggested that erythrocytes could accommodate enormous distortions depending on the dynamic dissociation of spectrin tetramers to dimers.^32^ Thus, distilled water easily breaks the cytoskeleton of native erythrocytes through rupture of spectrin tetramers caused by the hypotonic swelling (*t*_R_=0 min, Figure 7). At the beginning of the irradiation, UVA results in a small production of ROS, subsequently inducing some formation of Met-hemoglobins (Met-Hbs).^33^ Met-Hb can stabilize the skeleton against shear forces through promotion of head-to-head association between two spectrin dimers.^34,35^ We therefore propose that some Met-Hbs partly strengthen the spectrin-based cytoskeleton at *t*_R_=3 min, which permits influx of trypan blue but prevents outflow of Hbs (*t*_R_=3 min, Figure 7). Accordingly, our data show that UVA-pretreated cells, shaped as biconcave profile, were stained by trypan blue after addition of distilled water (Figure 2c). In addition, UVA pretreatment totally maintains the biconcave shape (Figure 5d) instead of spherical one (Figure 5b) that is observed in the presence of 0.0015% saponin. This confirms a strengthening of the cytoskeleton. With increasing irradiation time (dose), more Met-Hbs are produced. This leads to further enhancement of the cytoskeleton which completely prevents Hbs outflow and trypan blue entry even if distilled water is added (*t*_R_=5 min, Figure 7 and 2d). Hemin would be subsequently released from Hbs or Met-Hbs by means of ROS.^36^ Jarolim *et al.*^34^ also suggested that hemin induced dissociation of spectrins from actin junctional complexes by weakening the spectrin–protein 4.1–actin interaction, resulting in the loss of the stabilizing effect of Hbs on the cytoskeleton. Accordingly, we find that UVA-treated erythrocytes are marked again by trypan blue after application of distilled water (*t*_R_=6 min, Figure 7 and 2e). A strange point is that cells are marked by trypan blue in the outer ring at *t*_R_=5 min and 11 min. Based on the above analysis, we believe that some weakly scattered UVA light affects the cells in the outer ring, which provides a protective effect that is similar to that of 3 min UVA irradiation.

We also observed an autofluorescence change (450/58 nm emission filter) induced by 365 nm UVA (purple curve in Figure 7). Our previous work demonstrated that the change of autofluorescence coincides with ROS production because of the photodynamic effects.^37^ The purple curve shows that autofluorescence does not exhibit an evident increase because of the low fluorescence efficiency of Hb or Met-Hb at the beginning of the irradiation, implying low production of ROS. Thus, we believe that enhancement of the cytoskeleton makes a major contribution to shape protection in the first 6 mins although the ROS should affect the membrane. An increase of autofluorescence enhancement was observed 6 mins later. This is due to UVA-dependent production of bilirubin from degradation of hemin.^37^ Bilirubin, as an efficient photosensitizer, can generate an amount of ROS in the presence of UVA via PDT. In this case, the large number of ROS could lead to significant lipid peroxidation followed by membrane rigidification and shape change.^21,38,39^ As presented in this work, UVA irradiation can induce shrinkage of erythrocytes depending on the amount of ROS at *t*_R_=11 min and 20 min (blue curve in Figure 7 and 3e), which is similar to that of the membrane-soluble oxidant Cum-OOH (Figure 4e). The stiff lipid membrane instead of the cytoskeleton not only enables erythrocytes to maintain their biconcave profile against distilled water, but also block the entry of trypan blue (*t*_R_=11 min, Figure 7 and 3f). A recent paper has demonstrated that excessive lipid peroxidation could lead to pore formation.^24^ Correspondingly, as irradiation time increases further, trypan blue can directly permeate into erythrocytes without distilled water treatment (*t*_R_=20 min, Figure 7 and 3b). To resume, we suggest that UVA provides a periodical protective effect on the shape of erythrocytes through enhancement of the spectrin-based cytoskeleton and membrane rigidification sequentially.

In addition, an argument based on fixation by cross-linking the hemoglobin seem to clarify the observed effects resulted from UVA irradiation. Our explanation is shown as follow. First, the integrity of erythrocytes membrane tested by trypan blue changed periodically with increasing irradiation dose, which cannot be simply explained by monotonous fixation theory. It is known that at least two factors should contribute to the periodical changes. Second, our previous results^37,40^ and other researchers’ work^41^ demonstrated that UVA light could lead to hemoglobin decomposition rather than cross-linking. Meanwhile, as the important effector of UV irradiation, ROS was found to degrade hemoglobin.^36^ Furthermore, we employed two typical fixatives, paraformaldehyde (PFA, 4% for 20 min) and glutaraldehyde (GA, 0.1% for 20 min), to erythrocytes to simulate fixation. We found that Trypan blue could not stain PFA/GA-fixed erythrocytes (Supplementary Figures S2 and S3). Triton X-100 (0.5%, v/v) completely dissolved the PFA-fixed erythrocytes (Supplementary Figure S2d). In contrast, Triton had no dissolution effect on GA-treated erythrocytes (Supplementary Figure S3d). Meanwhile, trypan blue could not stain GA-fixed erythrocytes although Triton was used (Supplementary Figure S3e). For UVA treatment, Triton could not dissolve the UVA-treated erythrocytes (Supplementary Figure S4a). But, such erythrocytes were evidently stained by trypan blue (Supplementary Figure S4b). These data clearly showed that the fixation is different to that of UVA irradiation. Taken together, we believe that our theory of membrane-skeleton system enhancement are reasonable to explain the results.

In conclusion, 365 nm UVA irradiation can periodically protect the biconcave shape of human erythrocytes by means of enhancement of the membrane-skeleton system associated with a production of ROS in a dose-dependent manner. This stiff membrane-skeleton is accompanied by a loss of deformability. But all in all, our results provide a novel function for UVA as a method to protect the biconcave morphology of human erythrocytes, which might be of importance for clinical use of blood.

## Methods

### Ethics statement

The work was reviewed and approved by the Ethics Committee of Nankai University. All participants gave written informed consent. All methods were carried out in accordance with approved guidelines.

### Isolation of erythrocytes

Human erythrocytes were purified from the peripheral blood of healthy individuals by step-density gradient centrifugation over Histopaque 1119 solutions (11191, Sigma-Aldrich) at 500×*g* for 10 min. To preserve their biconcave shape, isolated erythrocytes were suspended in HBSS containing 1% (w/v) bovine serum albumin (V900933, Sigma-Aldrich) using stainless-steel chambers and then kept in a 4 °C refrigerator until use.^42,27^

### Irradiation system

The UVA irradiation system was based on an inverted fluorescence microscope (Axio observer D1, Carl Zeiss, Germany). The light from a 100 W mercury lamp, used as irradiation source, was passed through an attenuator and a 365/50 nm filter, subsequently focused on the samples by a Fluar ×40/1.30 oil UV objective. The irradiation area was determined by an aperture diaphragm. Irradiation time was controlled by a shutter (VS25S2ZM1, Uniblitz, USA) using MetaMorph software. Since cells were irradiated by UVA based on a microscope, we could clearly distinguish the irradiation and non-irradiation region at the single-cell level. The schematic diagram of the irradiation system is shown in Supplementary Figure S1a. In addition, by using rhodamine fluorescence imaging, we made sure that the distribution of the irradiation intensity was uniform (Supplementary Figures S2b and c).

### Microscopy

All bright-field observations were performed by an inverted microscope (Axio observer D1, Carl Zeiss), and recorded by a color CCD (MicroPublisher 5.0, Qimaging, Canada). Panorama images were assembled from a series of images and combined by a panoramic stitching software (PTGui, New House Internet Services BV, Rotterdam, Netherlands). For fluorescent imaging, erythrocytes were incubated with 300 nM nile red (a hydrophobic probe for membrane detection) at room temperature for 20 min in HBSS. Then experiments were performed using an inverted confocal microscope (LSM700, Carl Zeiss, Germany) with a ×63 oil objective. 3D images were reconstructed from Z-stacks with optical sections of 500 nm using the ImageJ software.
