# Supplementary material for: Protection of the biconcave profile of human erythrocytes against osmotic damage by ultraviolet-A irradiation through membrane-cytoskeleton enhancement
Source: Cell Death Discov. 2017 Jul 17;3:17040–. doi: 10.1038/cddiscovery.2017.40 (PMC5512140; doi:10.1038/cddiscovery.2017.40)

**Protection of the biconcave profile of human erythrocytes against distilled water by ultraviolet-A irradiation**

Cunbo Li^1,6^, Zheming Li^1,6^, Shuang Xun^1^, Pengchong Jiang^1^, Rui Yan^2^, Mincai Chen^3^, Fen Hu^1^, Romano A. Rupp^1^, Xinzheng Zhang^1^, Leiting Pan^1,4^,*, Jingjun Xu^1,5^

*^1^Key Laboratory of Weak-Light Nonlinear Photonics, Ministry of Education, TEDA Institute of Applied and Physics School of Physics, Nankai University, Tianjin, China*

*^2^Department of Chemistry, University of California, Berkeley, Berkeley, California, USA*

*^3^Department of Blood Transfusion, PLA 307 Hospital, Beijing, China*

*^4^The 2011 Project Collaborative Innovation Center for Biological Therapy, Nankai University, Tianjin, China*

*^5^Collaborative Innovation Center of Extreme Optics, Shanxi University, Taiyuan, Shanxi, China*

**Supplementary Movie** **S1** A micropipette is used to detect the deformation of native erythrocytes based on siphonage.

**Supplementary Movie** **S2** A micropipette is used to detect the deformation of erythrocytes based on siphonage. Cells are treated with UVA for 1 min.

**Supplementary Movie S3** A micropipette is used to detect the deformation of erythrocytes based on siphonage. Cells are treated with UVA for 2 min.

**Supplementary Movie** **S4** A micropipette is used to detect the deformation of erythrocytes based on siphonage. Cells are treated with UVA for 5 min.

**Supplementary Figure S1** **365 nm UVA irradiation system at single cell-level**. (**a**) Schematic diagram of the UVA irradiation system based on an inverted fluorescence microscope. (**b**) Fluorescence image of rhodamine. (**c**) The spatially homogeneous distribution of the rhodamine fluorescence along the horizontal direction indicates a uniform distribution of the excitation light.


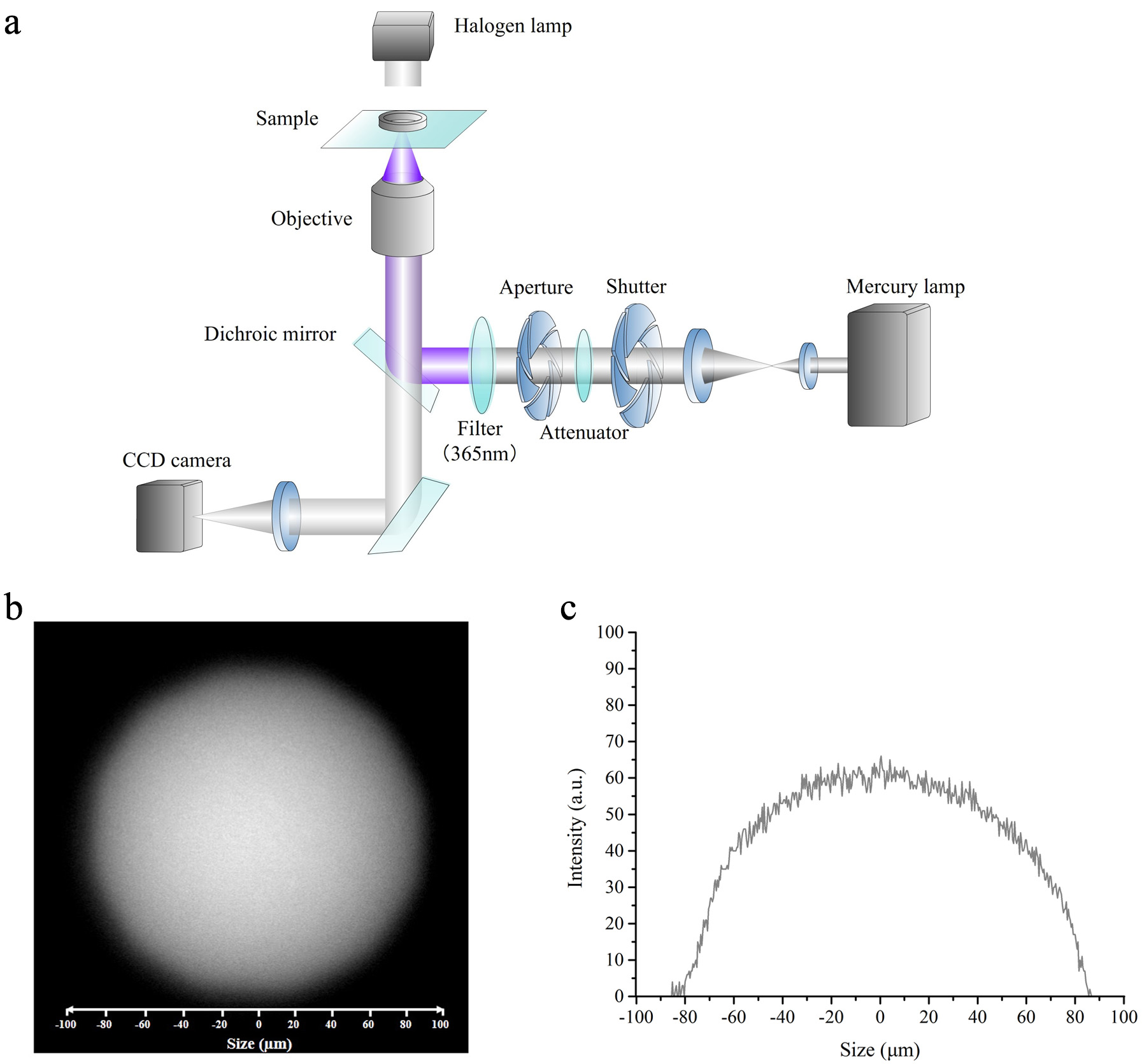


**Supplementary Figure** **S2** **The effect of PFA fixation on the profile of human erythrocytes**. (**a**) Normal erythrocytes with typical biconcave shape. (**b**) Cells were fixed by 4% PFA UVA for 20 min. (**c**) Trypan blue could not stain erythrocytes after treatment of PFA. (**d**) 0.5% Triton X-100 turned erythrocytes into ghosts even though cells were fixed by PFA.


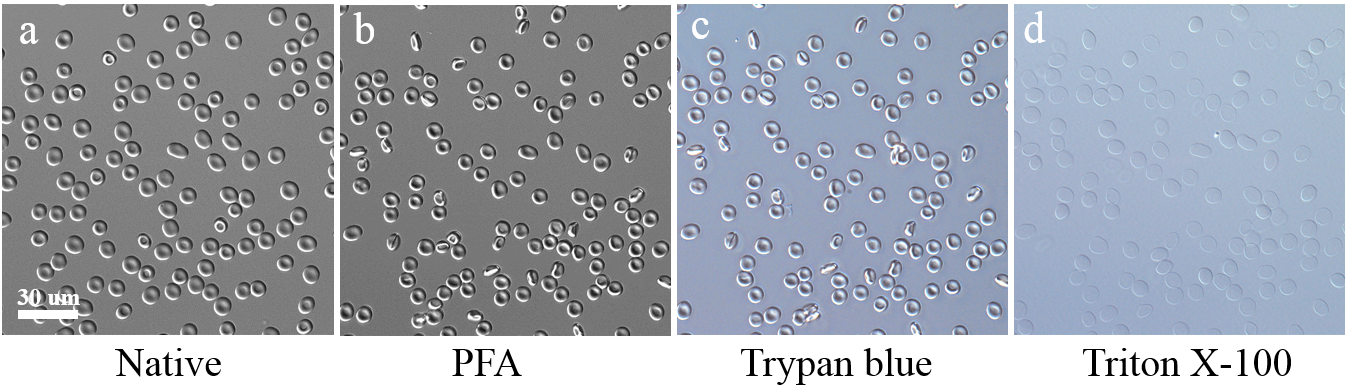


**Supplementary Figure** **S3** **The effect of GA fixation on the shape of human erythrocytes**. (**a**) Normal erythrocytes with typical biconcave profile. (**b**) Cells were fixed by 0.1% GA for 20 min. (**c, e**) Trypan blue could not stain GA-fixed erythrocytes no matter whether Triton is used or not. (**d**) 0.5% Triton X-100 had no dissolution effect on GA-treated erythrocytes.


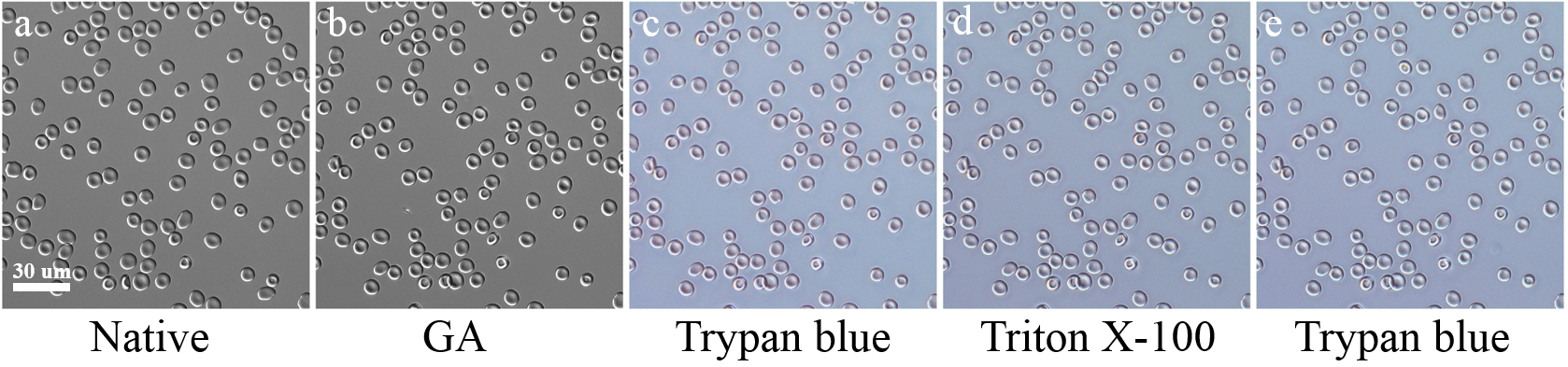


**Supplementary Figure** **S4** **The effect of Triton on the profile of UVA-pretreated human erythrocytes**. (**a**) UVA irradiation protected the biconcave shape of human erythrocytes (inside of the red circle) against 0.5% Triton X-100. (**b**) UVA-pretreated erythrocytes could be stained by trypan blue after application of Triton X-100.


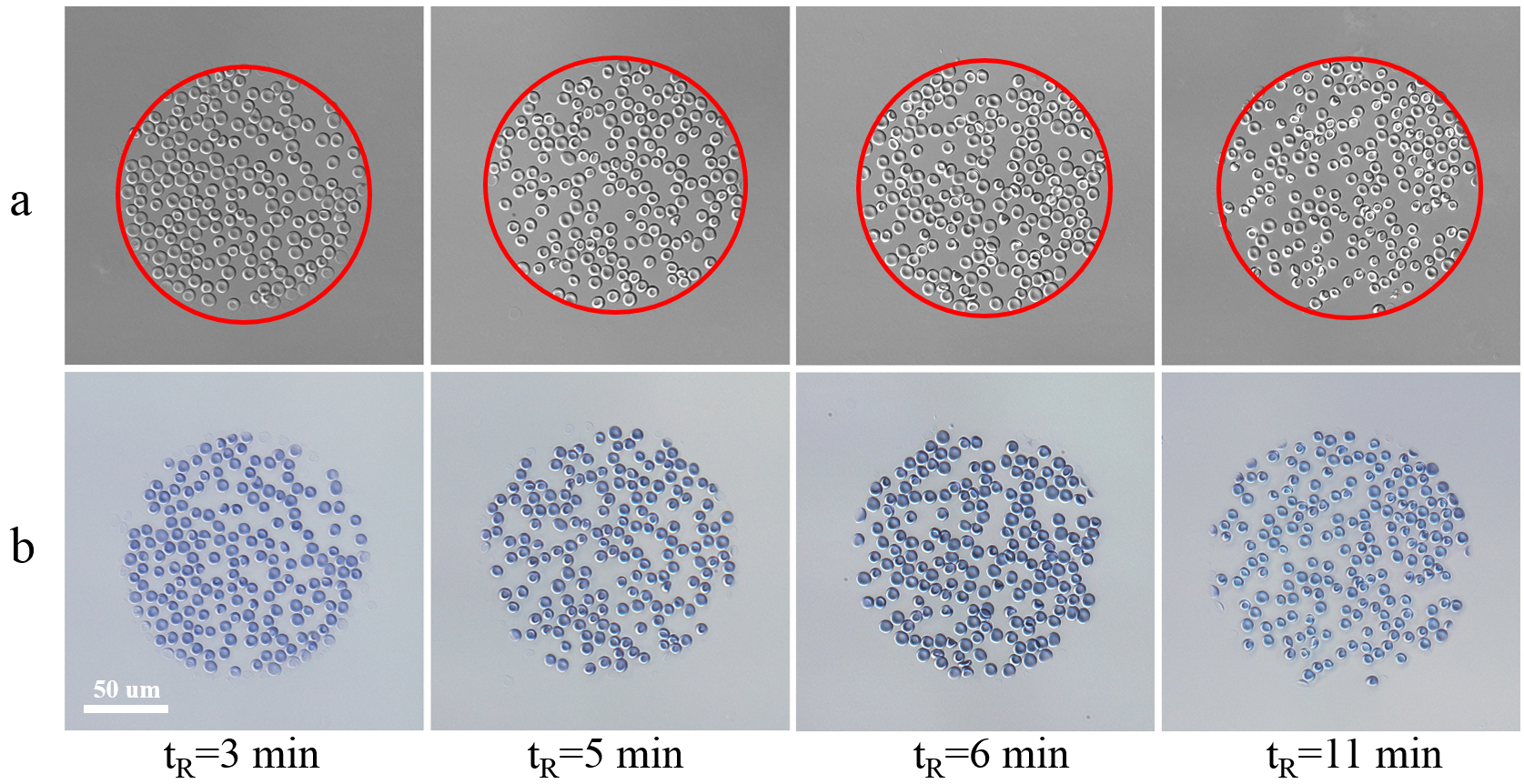

Supplement: Supplementary Information [file cddiscovery201740-s1.doc]
